# Relationship between chronic pain syndrome and anxiety disorders in patients with rheumatoid arthritis

**DOI:** 10.1192/j.eurpsy.2022.1701

**Published:** 2022-09-01

**Authors:** N. Chernus, R. Gorenkov, S. Sivkov, A. Sivkov, T. Savina, A. Serdakova, A. Zolotovickaja

**Affiliations:** The I.M. Sechenov First Moscow State Medical University: Moscow, Russia, The Outpatient Care Department, Moscow, Russian Federation

**Keywords:** pain, anxiety, rheumatoid arthritis, pain,

## Abstract

**Introduction:**

Chronic pain syndrome is still one of the leading complaints of patients with rheumatoid arthritis (RA).

**Objectives:**

Study the relationship between chronic pain syndrome of different duration and the level of anxiety disorders.

**Methods:**

Clinical and psychophysiological examination of 76 patients with RA was carried out, the average age was 42.4 ± 7.2 years. The severity of pain syndrome was determined on the VAS scale, the level of anxiety by the Spielberger-Hanin technique

**Results:**

Analysis of pain syndrome according to YOUR revealed higher rates (p < 0, () 1) in patients with shorter periods of disease: up to 12 months and more than 12 months: 66.0 ± 1.5 mm and 61.9 ± 1.5 mm, respectively, than in patients with a longer period of war - more than 3 years (53.7 ± 1.0 mm). Psychophysiological examination of RA patients revealed anxiety spectrum disorders in 53 (69.7%) patients. The severity of anxiety disorders was different depending on the duration of the chronic pain syndrome: the highest indicators of reactive anxiety were detected in patients with a length of pain syndrome of up to 12 months: 45.7 ± 0.6 points, in patients with a disease period of more than 12 months - 42.4 ± 0.5 points, and in patients with a disease period of more than 3 years 37.6 + 0.5 points.

**Conclusions:**

Thus, a direct correlation between the degree of pain severity and the level of anxiety disorders is revealed, which is desirable to consider when selecting pathogenetic therapy

**Disclosure:**

No significant relationships.

